# Longitudinal association between statins and changes in CT‐derived body composition in patients with abdominal aortic aneurysm

**DOI:** 10.1002/jcsm.13565

**Published:** 2025-04-09

**Authors:** Nicholas A. Bradley, Amy Walter, Chiara Sankey, Alasdair Wilson, Tamim Siddiqui, Campbell S.D. Roxburgh, Donald C. McMillan, Graeme J.K. Guthrie

**Affiliations:** ^1^ University of Glasgow Glasgow UK; ^2^ NHS Tayside Dundee UK; ^3^ NHS Grampian Aberdeen UK; ^4^ NHS Lanarkshire East Kilbride UK

**Keywords:** AAA, body composition analysis, EVAR, OSR, sarcopenia, statin, systemic inflammation

## Abstract

**Background:**

Loss of skeletal muscle mass and systemic inflammation may offer prognostic value in patients with abdominal aortic aneurysm (AAA). The longitudinal progression of abnormal body composition parameters and their determinants is poorly reported. Statins are widely used medications that improve the prognosis of cardiovascular disease and interact with both muscle tissue and systemic inflammation. The present study aimed to describe the association between statin therapy and both pre‐operative and longitudinal CT‐derived body composition in patients undergoing elective intervention for AAA.

**Methods:**

A total of 756 consecutive patients undergoing elective intervention for AAA at three centres were retrospectively recruited. Body composition analysis was performed on pre‐operative and follow‐up CTs at L3 to generate subcutaneous adipose tissue index, visceral adipose tissue index and skeletal muscle index and density (SMI and SMD). Systemic inflammation was assessed using the systemic inflammatory grade.

**Results:**

A total of 756 patients (702 [93%] males, median [interquartile range, IQR] age 73.0 [11.0] years) were included, with a median (IQR) follow‐up of 67.0 (32) months and 235 deaths during the follow‐up period. There were 582 patients (77%) receiving statin therapy and 174 patients (23%) not receiving statin therapy. Follow‐up CTs were available for 273 patients. From pre‐operative to follow‐up CTs, there was a decrease in median SMI (*P* < 0.001) and SMD (*P* < 0.001) and an increase in the comparative prevalences of low SMI (43% vs. 50%, *P* < 0.01) and low SMD (64% vs. 88%, *P* < 0.001). There were no differences in baseline clinicopathological characteristics, systemic inflammation or pre‐operative CT‐derived body composition parameters between patients with and without >10% loss of skeletal muscle mass. In patients with ≤10% loss of SMI, mean (95% confidence interval) survival was 91.6 (87.2–95.9) months versus 89.3 (80.4–98.2) months in patients with >10% loss of SMI (*P* = 0.58). Patients receiving statin therapy had a higher American Society of Anesthesiologists grade (*P* < 0.001), a higher body mass index (BMI) (*P* < 0.05) and a greater prevalence of normal pre‐operative SMI (*P* < 0.001).

**Conclusions:**

In patients with AAA, skeletal muscle mass and density appear to progressively decline despite treatment of AAA, though specific determinants of this are uncertain, and statin use does not appear to predispose to either muscle loss or preservation. Statin therapy appears to be associated with a lower rate of pre‐operative low skeletal muscle mass, despite greater comorbidity and BMI. Further investigation of the progressive changes in muscle mass and quality, statin therapy and systemic inflammation is warranted.

## Introduction

Abdominal aortic aneurysm (AAA), a condition characterized by pathological dilatation of the aorta, is common in the UK population, in particular in males over the age of 65.^1^ The risk of aneurysm rupture is largely dictated by increasing size, and typical contemporary UK practice is to consider intervention in AAA over 5.5 cm.^2^ Intervention may be either open surgical repair (OSR) or endovascular aneurysm repair (EVAR), with ‘complex’ aneurysms repaired using fenestrated and branched endografts (F/B‐EVAR).

Low skeletal muscle mass and density, as assessed by CT‐derived body composition (CT‐BC) analysis, have been described in relation to a range of disease states and appear to confer an inferior prognosis.^3^, ^4^ Whilst predictors of low skeletal muscle mass in the pre‐operative period have been extensively investigated, factors associated with the rate of skeletal muscle loss through longitudinal analyses are less well described. Several studies in patients with and without cardiovascular disease report longitudinal analyses.^5^, ^6^ It remains uncertain whether either disease‐specific or host‐specific factors dominate the development and progression of low skeletal muscle mass, with recent data suggesting that host factors may play a greater role than previously thought.^7^ The current lack of longitudinal CT‐BC data limits our understanding of the natural history of skeletal muscle loss even when the disease with which it may be associated is treated, for example, repair of AAA or cancer resection.

Activation of the systemic inflammatory response (SIR) is increasingly being recognized as a key aetiological component of cardiovascular disease.^8^ Quantification of the SIR can be performed using the systemic inflammatory grade (SIG), which combines the acute phase (modified Glasgow prognostic score [mGPS] based on C‐reactive protein [CRP] and albumin) and differential white cell (neutrophil:lymphocyte ratio [NLR] based on absolute neutrophil and lymphocyte count) responses to give a comprehensive measure of the SIR.^9^, ^10^ Elevated SIG appears to be associated both with loss of skeletal muscle mass and density and independently with an inferior prognosis in patients with a range of conditions.^11^, ^12^, ^13^

Statins, HMG‐CoA reductase inhibitors, are one of the most commonly prescribed medications in high‐ and middle‐income countries.^14^ Their predominant role is in the primary and secondary prevention of cardiovascular disease, largely thought to be due to their lipid‐lowering effect. Guidelines recommend prescribing statins to patients with AAA on this basis.^15^ More recently, the effect of statins as anti‐inflammatory medications has become more widely reported, and the anti‐inflammatory effect has been explored as a potential therapeutic mechanism.^16^, ^17^ The major side effect of statins, reported in up to 15% of patients, is myalgia, with the underlying myopathic mechanism poorly understood.^18^, ^19^ Somewhat paradoxically, a small series of patients with cardiovascular disease have emerged describing an association between statin therapy, increased baseline skeletal muscle mass^20^ and a reduced rate of skeletal muscle loss.^21^ In patients with cardiovascular disease, studies describing longitudinal CT‐BC data and those describing the role of statin therapy are relatively small and, in some cases, report non‐standard measures of CT‐BC parameters.

The present study aimed to describe the association between pre‐operative and longitudinal CT‐BC parameters, statin therapy, systemic inflammation and survival in a cohort of patients undergoing elective intervention for AAA.

## Patients and methods

### Patient selection

Patients were retrospectively identified from theatre records at three large tertiary referral centres in Scotland, UK, representing cases drawn from three health boards (NHS Grampian, NHS Lanarkshire and NHS Tayside). Due to the retrospective, exploratory nature of the study design, formal sample size calculations were not performed. Specific procedural techniques were at the discretion of each institution, though practice was broadly similar between sites throughout the study period. Consecutive cases undergoing elective EVAR, F/B‐EVAR or OSR to treat aortic aneurysmal disease between 01/01/2015 and 10/01/2021 were screened for inclusion. Patients with active malignancy, active infection, isolated iliac aneurysms, aortic dissection, penetrating aortic ulcer, incomplete clinical or follow‐up data or emergency/urgent cases were excluded. Additionally, patients with corrupted or unavailable CT images, which precluded analyses, were excluded. Clinical, demographic and comorbidity data were recorded from electronic case records and patients' community health records. Age (≤75 and >75 years) and body mass index (BMI) (<25 and ≥25 kg/m^2^) were considered as categorical variables. Comorbidity was assessed using ASA (American Society of Anesthesiologists), which was recorded from operative records and subgrouped (≤2 or >2) in keeping with previous literature.^22^ Socio‐economic deprivation was assessed using the Scottish Index of Multiple Deprivation (SIMD), a government‐led postcode‐based measure of deprivation, with patients classified into deciles and then grouped on the basis of below (‘more deprived’) and above (‘less deprived’) the median decile. The minimum follow‐up interval for survival analysis was 2 years from the date of surgery. Survival data were obtained from the Community Health Index (CHI) registry, which is routinely available and maintained at a national health board level and populated from both primary and secondary care data; however, cause of death data were not available from this registry. West of Scotland Research Ethics Committee approval was obtained for this study (Reference 21/WS/0146; approval granted 23/11/2021).

### Prescribing data

Data on pre‐operative medications were recorded from pre‐assessment clinic data, which are routinely performed in all patients prior to major elective intervention. This constitutes a structured assessment that is performed by a specialist nurse practitioner, where accurate current prescribing data is recorded from both primary care records and directly from the patient. The specific choice of statin therapy was at the discretion of the primary care team based on their local policies, and typical practice in patients with AAA is to prescribe the maximum tolerated dose. Patients were subgrouped by statin use (yes/no), and outcomes were compared between subgroups.

### Pre‐operative CT images

Contrast‐enhanced arterial‐phase CTs performed routinely as part of the pre‐operative workup were accessed, and a slice at the midpoint of the L3 vertebral body (identified by anatomical landmarks) was chosen for analysis.

### Follow‐up CT images

Longitudinal analyses of follow‐up contrast‐enhanced arterial‐phase CT images at L3 in patients undergoing EVAR or F/B‐EVAR (OSR patients do not typically undergo surveillance) were performed. Surveillance protocols were different between institutions (with some preferring ultrasound surveillance); however, the typical policy was to perform a follow‐up CT at approximately 12 months post‐procedure. Medical records of all patients undergoing EVAR or F/B‐EVAR were screened, and CTs performed ≥11 months post‐procedure were included, with no maximal time interval (range 11–71 months). Cases were excluded if the CT was performed for non‐surveillance indications, if follow‐up CT identified an incidental confounding pathology (e.g., a new radiological diagnosis of cancer) or if a new confounding pathology was documented in the medical records between the index procedure and the follow‐up scan. Cases were not excluded on the basis of endoleak, as this was thought unlikely to influence CT‐BC.

### Body composition analysis

Body composition analysis was performed using established methodology^23^ by two authors (N. A. B. and C. S.), with an intraclass correlation coefficient of 0.94 indicating excellent correlation. Subcutaneous adipose tissue, visceral adipose tissue, skeletal muscle area (SMA) and skeletal muscle density (SMD) were manually measured using ImageJ v1.53 software using muscle tissue thresholds of −29 to +150 Hounsfield units (HU) and adipose tissue thresholds of −190 to −30 HU. The areas obtained were normalized to height^2^ to generate subcutaneous adipose tissue and visceral adipose tissue indices (SATI and VATI) and skeletal muscle index (SMI), whilst SMD was not normalized. Sex‐specific thresholds of these CT‐BC parameters previously derived from a prior reported cohort of patients with AAA^23^ were applied to each parameter to subgroup patients into ‘normal’ and ‘high/low’. Additionally, for follow‐up CTs, the ΔSMI (cm^2^/m^2^) was calculated as SMI_follow‐up_ − SMI_pre‐op_. The %ΔSMI was then calculated as ΔSMI/SMI_pre‐op_ and normalized to the interval from baseline CT to surveillance CT to generate %ΔSMI/year. This method was repeated for SATI, VATI and SMD. Patients were also subgrouped based on SMI or SMD change (≤10% or >10% loss in keeping with previous longitudinal CT‐BC analyses^5^), and comparisons between groups were made.

### Inflammatory profiling

Institutional policy during the study period was to admit patients to the hospital on the evening prior to surgery, where pre‐operative blood work was routinely performed as part of existing patient care. mGPS and NLR were then combined into SIG, as previously reported.^9^, ^10^ Outcomes were compared between groups of SIG 0 (considered ‘non‐inflamed’) versus SIG 1 (considered ‘mildly inflamed’) versus SIG ≥ 2 (considered ‘inflamed’).

### Outcomes of interest

The outcomes of interest were the association between statin therapy and pre‐operative CT‐BC parameters, the difference between pre‐operative and follow‐up CT‐BC parameters and the different factors associated with >10% or ≤10% skeletal muscle loss from pre‐operative to follow‐up CTs.

### Statistical analyses

Differences between unpaired continuous variables were compared by the Mann–Whitney test and between unpaired categorical variables using the *χ* ^2^ test (linear‐by‐linear *P* values reported). Differences between paired continuous variables were compared by the Wilcoxon matched‐pairs test and between paired categorical variables using the McNemar test. Time‐to‐event analyses were calculated using the Kaplan–Meier method, with differences between cohorts assessed using the log‐rank *t*‐test. Where time‐to‐event survival data did not reach a median survival, the mean (95% confidence interval [CI]) values are reported. The association between variables and overall survival was assessed using a Cox proportional hazards model. The association between covariates and loss of SMI or SMD was analysed using binary logistic regression. In all regression models, variables were initially interrogated in univariate analysis, and those with a univariate *P* < 0.05 were included in a multivariate model. Missing data were selectively excluded from relevant analyses on a case‐by‐case basis. Typically, missing CT‐BC parameters were due to CT image compromise or missing height, and missing SIG was due to a lack of CRP on pre‐operative blood work. Due to the wide range in follow‐up CT intervals, a sensitivity analysis only on CTs performed 11–18 months following intervention was performed. All analyses were performed using IBM SPSS 28.0. *P* values < 0.05 were considered statistically significant.

## Results

A total of 756 patients undergoing elective intervention for AAA were eligible for inclusion in the study. There were 154 (20%) patients treated by OSR, 702 (93%) males and a median (interquartile range [IQR]) age of 73.0 (11.0) years. The median (IQR) follow‐up was 75 (32) months, and there were 272 (36%) deaths during the follow‐up period. The mean (95% CI) survival in the entire study population was 90.0 (84.0–96.1) months. Regarding longitudinal analyses, there were 599 patients who underwent EVAR or F/B‐EVAR with suitable pre‐operative CTs for comparison, of which 273 (46%) had a suitable follow‐up CT and were included in longitudinal analyses. The median (IQR) time from procedure to follow‐up CT was 15 (9) months. *Table* *1* displays the differences between patients who underwent EVAR or F/B‐EVAR with (*n* = 273) and without (*n* = 326) follow‐up CTs. Patients with follow‐up CTs were typically younger (*P* = 0.04), with lower socio‐economic deprivation (*P* = 0.03) and a lower prevalence of pre‐operative anaemia (*P* < 0.01).

**Table 1:** The difference between baseline clinical characteristics, systemic inflammation and pre‐operative CT‐derived body composition parameters between patients with and without per‐protocol follow‐up CTs in a cohort of patients undergoing elective EVAR or F/B‐EVAR for AAA (*n* = 599)

	Follow‐up CT (*n* = 273)	No follow‐up CT (*n* = 326)	*P*
Age (years)			
<65	16 (6%)	15 (5%)	
65–75	151 (55%)	157 (48%)	
>75	105 (39%)	154 (47%)	**0.04**
Sex			
Male	254 (93%)	297 (91%)	
Female	19 (7%)	29 (9%)	0.39
ASA			
≤2	154 (57%)	191 (59%)	
>2	118 (43%)	134 (41%)	0.60
BMI (kg/m^2^)			
<25	53 (19%)	67 (21%)	
≥25	220 (81%)	258 (79%)	0.72
SIMD			
More deprived	118 (43%)	170 (52%)	
Less deprived	154 (57%)	156 (48%)	**0.03**
Repair strategy			
F/B‐EVAR	53 (19%)	76 (23%)	
EVAR	220 (81%)	250 (77%)	0.25
Baseline AAA diameter (mm)			
<65	196 (73%)	233 (72%)	
≥65	74 (27%)	90 (28%)	0.90
Statin use			
Yes	218 (80%)	251 (77%)	
No	55 (20%)	75 (23%)	0.40
Baseline creatinine			
Normal	208 (77%)	236 (73%)	
High	62 (23%)	89 (27%)	0.22
Baseline haemoglobin			
Normal	227 (84%)	239 (74%)	
Low	43 (16%)	86 (26%)	**<0.01**
SIG			
0	134 (56%)	137 (52%)	
1	72 (30%)	83 (32%)	
≥2	33 (14%)	43 (16%)	0.32
Pre‐operative SATI			
Normal	31 (12%)	38 (12%)	
High	224 (88%)	265 (88%)	0.81
Pre‐operative VATI			
Normal	67 (25%)	79 (25%)	
High	106 (75%)	241 (75%)	0.97
Pre‐operative SMI			
Normal	156 (57%)	167 (54%)	
Low	117 (43%)	142 (46%)	0.45
Pre‐operative SMD			
Normal	98 (36%)	97 (31%)	
Low	175 (64%)	218 (69%)	0.19

*Note*: *P* values generated through linear‐by‐linear *χ* ^2^ analyses comparing the proportion of each covariate within each subgroup. Bold values denote statistically significant values (*P* < 0.05). Abbreviations: AAA, abdominal aortic aneurysm; ASA, American Society of Anesthesiologists grade; BMI, body mass index; EVAR, endovascular aneurysm repair; F/B‐EVAR, fenestrated/branched EVAR; SATI, subcutaneous adipose tissue index; SIG, systemic inflammatory grade; SIMD, Scottish Index of Multiple Deprivation; SMD, skeletal muscle density; SMI, skeletal muscle index; VATI, visceral adipose tissue index.

The characteristics of the entire study cohort when subgrouped by statin therapy are shown in *Table* *2*. There were 582 patients (77%) receiving statin therapy and 174 patients (23%) not receiving statin therapy, and during follow‐up, there were 206 (35%) deaths in the statin therapy subgroup and 66 (38%) deaths in the no statin therapy subgroup. Patients receiving statin therapy had a higher ASA (*P* < 0.001) and a higher BMI (*P* = 0.01). Regarding CT‐BC, high SATI (*P* = 0.04) and high VATI (*P* < 0.01) were more prevalent in patients receiving statin therapy, whilst low SMI (*P* < 0.001) was less prevalent in patients receiving statin therapy. On univariate analysis of patients taking statins, higher age (*P* < 0.001), high creatinine (*P* = 0.01), low haemoglobin (*P* < 0.01), low pre‐operative SMI (*P* < 0.001) and low pre‐operative SMD (*P* < 0.01) were associated with inferior survival, whilst high BMI (*P* < 0.01), high pre‐operative SATI (*P* < 0.01) and high pre‐operative VATI (*P* = 0.03) were associated with superior survival. On multivariate analysis of patients taking statins, higher age (hazard ratio [HR] 1.72, 95% CI 1.31–2.26, *P* < 0.001), high pre‐operative SATI (HR 0.59, 95% CI 0.37–0.95, *P* = 0.04) and low pre‐operative SMD (HR 1.75, 95% CI 1.23–2.49, *P* < 0.01) were associated with survival. On univariate analysis of patients not taking statins, higher age (*P* < 0.001), higher AAA diameter (*P* = 0.03), high creatinine (*P* < 0.01), low haemoglobin (*P* < 0.001), low pre‐operative SMI (*P* < 0.001) and low pre‐operative SMD (*P* = 0.01) were associated with inferior survival, whilst OSR (*P* < 0.01) was associated with superior survival. On multivariate analysis of patients not taking statins, higher age (HR 1.77, 95% CI 1.05–3.00, *P* = 0.04) and low pre‐operative SMI (HR 2.71, 95% CI 1.44–5.10, *P* < 0.01) were associated with survival.

**Table 2:** The difference between demographic and clinicopathological characteristics, CT‐BC, systemic inflammation and the association between demographic and clinicopathological characteristics and survival in patients undergoing elective EVAR or OSR for AAA subgrouped by statin therapy (*n* = 756)

	Statin therapy (*n* = 582)	No statin therapy (*n* = 174)	*P* ^a^	Statin therapy (*n* = 582)		No statin therapy (*n* = 174)	
				Univariate^b^	Multivariate^b^	Univariate^b^	Multivariate^b^
Age (years)							
<65	42 (7%)	10 (6%)	0.53	2.03	1.72	2.70	1.77
65–75	313 (54%)	93 (53%)		1.59–2.60	1.31–2.26	1.72–4.25	1.05–3.00
>75^c^	227 (39%)	71 (41%)		**<0.001**	**<0.001**	**<0.001**	**0.04**
Sex				1.19		0.71	
Male	538 (92%)	162 (93%)	0.77	0.73–1.93	—	0.22–2.28	—
Female^c^	44 (8%)	12 (7%)		0.48		0.57	
ASA				1.10		1.57	
≤2	298 (51%)	117 (68%)	**<0.001**	0.84–1.45	—	0.95–2.59	—
>2^c^	281 (49%)	56 (32%)		0.49		0.08	
BMI (kg/m^2^)				0.63	1.08	1.11	
<25	109 (19%)	47 (27%)	**0.01**	0.45–0.86	0.68–1.70	0.64–1.94	—
≥25^c^	470 (81%)	126 (73%)		**<0.01**	0.76	0.71	
SIMD				0.85		0.87	
More deprived^c^	279 (48%)	71 (41%)	0.09	0.65–1.13	—	0.53–1.45	—
Less deprived	300 (52%)	103 (59%)		0.26		0.60	
Repair strategy				0.84		0.33	0.50
EVAR or F/B‐EVAR	473 (81%)	131 (75%)	0.08	0.58–1.23	—	0.15–0.73	0.21–1.15
OSR^c^	109 (19%)	43 (25%)		0.37		**<0.01**	0.10
AAA diameter (mm)				1.19		1.85	1.34
<65	419 (73%)	123 (72%)	0.80	0.89–1.59	—	1.12–3.06	0.76–2.37
≥65^c^	159 (27%)	49 (28%)		0.25		**0.03**	0.32
Baseline creatinine				1.48	1.18	2.20	1.54
Normal	447 (77%)	129 (75%)	0.52	1.10–2.00	0.85–1.65	1.31–3.69	0.86–2.77
High^c^	131 (23%)	43 (25%)		**0.01**	0.33	**<0.01**	
Baseline Hb				1.52	1.18	2.60	1.63
Normal	461 (80%)	142 (83%)	0.34	1.11–2.09	0.84–1.66	1.50–4.50	0.85–3.12
Low^c^	117 (20%)	29 (17%)		**<0.01**	0.35	<**0.001**	0.15
SIG							
0	262 (53%)	66 (48%)		1.12		1.40	
1	158 (32%)	49 (36%)		0.92–1.37	—	0.96–2.03	—
≥2^c^	71 (15%)	22 (16%)	0.34	0.25		0.08	
Pre‐operative SATI				0.54	0.59	0.91	
Normal	61 (11%)	28 (17%)	**0.04**	0.37–0.78	0.37–0.95	0.47–1.76	—
High^c^	480 (89%)	135 (83%)		**<0.01**	**0.04**	0.79	
Pre‐operative VATI				0.70	0.73	1.04	
Normal	141 (25%)	61 (36%)	**<0.01**	0.52–0.95	0.51–1.04	0.62–1.74	—
High^c^	434 (75%)	111 (64%)		**0.03**	0.09	0.89	
Pre‐operative SMI				1.61	1.22	3.35	2.71
Normal	338 (60%)	74 (44%)	**<0.001**	1.22–2.12	0.90–1.67	1.84–6.07	1.44–5.10
Low^c^	226 (40%)	96 (56%)		**<0.001**	0.21	**<0.001**	**<0.01**
Pre‐operative SMD				1.71	1.75	1.85	1.49
Normal	195 (34%)	68 (40%)		1.24–2.35	1.23–2.49	1.06–3.22	0.82–2.70
Low^c^	376 (66%)	104 (60%)	0.20	**<0.01**	**<0.01**	**0.01**	0.19

*Note*: Bold values denote statistically significant values (*P* < 0.05). Abbreviations: AAA, abdominal aortic aneurysm; ASA, American Society of Anesthesiologists grade; BMI, body mass index; EVAR, endovascular aneurysm repair; F/B‐EVAR, fenestrated/branched EVAR; SATI, subcutaneous adipose tissue index; SIG, systemic inflammatory grade; SIMD, Scottish Index of Multiple Deprivation; SMD, skeletal muscle density; SMI, skeletal muscle index; VATI, visceral adipose tissue index.

^a^ *P* values generated through linear‐by‐linear *χ* ^2^ analyses comparing proportions of each variable between the statin therapy (yes/no) subgroups.

^b^ Data presented as hazard ratio (HR), 95% confidence interval and *P* value, generated through the Cox proportional hazards model testing the association between variables and overall survival.

^c^ The category the HR pertains to in relation to the reference category.

**Figure 1:**
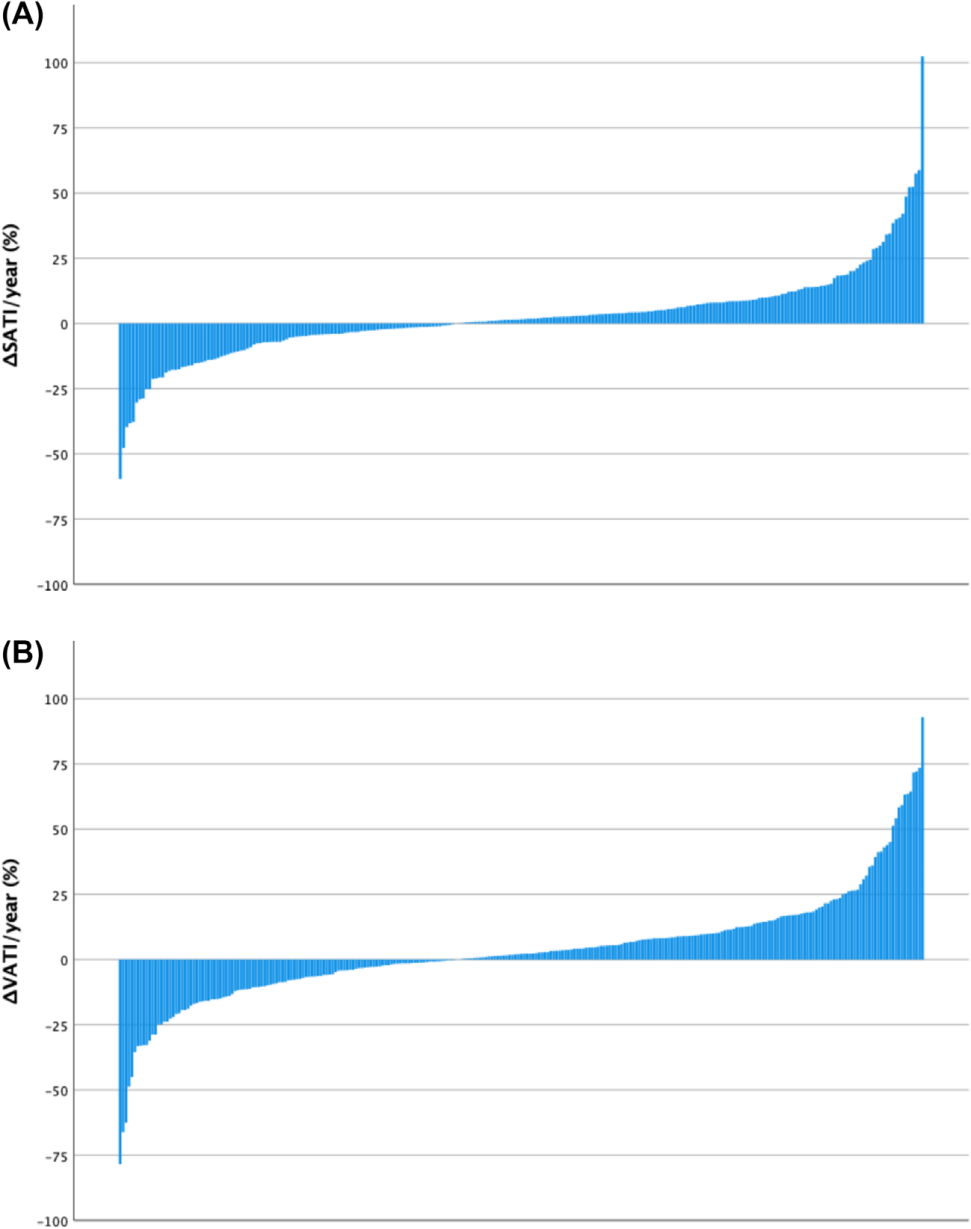
Percentage change in (A) subcutaneous adipose tissue index (ΔSATI) and (B) visceral adipose tissue index (ΔVATI) per year between pre‐operative and follow‐up CTs in patients undergoing elective endovascular repair of abdominal aortic aneurysm (*n* = 273).

**Figure 2:**
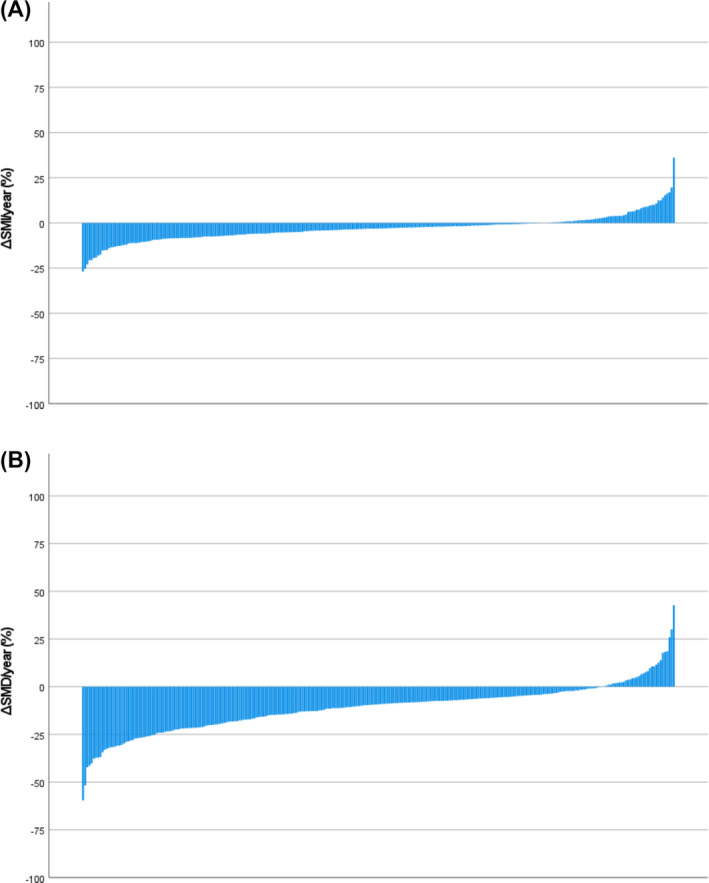
Percentage change in (A) skeletal muscle index (ΔSMI) and (B) skeletal muscle density (ΔSMD) per year between pre‐operative and follow‐up CTs in patients undergoing elective endovascular repair of abdominal aortic aneurysm (*n* = 273).

*Table* *3* displays the differences between pre‐operative and follow‐up categorical CT‐BC parameters in the entire longitudinal cohort (*n* = 273) and in patients subgrouped by statin therapy. In the entire longitudinal cohort, from pre‐operative to follow‐up CTs, the comparative prevalences of high SATI (*P* = 0.63) and high VATI (*P* = 0.22) were similar, and there was an increase in the comparative prevalences of low SMI (43% vs. 50%, *P* < 0.01) and low SMD (64% vs. 88%, *P* < 0.001). These changes are also displayed in the waterfall plots in *Figures* *1* and *2*, where %ΔSATI/VATI/SMI/SMD per year is displayed. When subgrouped by statin therapy (statin therapy *n* = 218, no statin therapy *n* = 55), from pre‐operative to follow‐up CTs, there was no change in SATI or VATI in patients both taking and not taking statins. In patients taking statins, there was an increase in the prevalence of low SMI (38% vs. 45%, *P* = 0.01), and there was an increase in the prevalence of low SMD in patients both taking (64% vs. 89%, *P* < 0.001) and not taking (62% vs. 84%, *P* < 0.001) statins.

**Table 3:** The comparison of pre‐operative and longitudinal CT‐derived body composition parameters (categorical) in patients with per‐protocol follow‐up CTs undergoing elective EVAR or F/B‐EVAR for AAA subgrouped by statin use (*n* = 273)

	Entire longitudinal cohort (*n* = 273)			Statin therapy (*n* = 218)			No statin therapy (*n* = 55)		
	Pre‐operative CT	Follow‐up CT	*P*	Pre‐operative CT	Follow‐up CT	*P*	Pre‐operative CT	Follow‐up CT	*P*
SATI									
Normal SATI	31 (12%)	22 (13%)		19 (9%)	21 (10%)		12 (24%)	12 (22%)	
High SATI	224 (88%)	230 (87%)	0.63	186 (91%)	188 (90%)	0.58	38 (76%)	42 (78%)	1.00
VATI									
Normal VATI	67 (25%)	59 (22%)		49 (23%)	44 (20%)		18 (33%)	15 (27%)	
High VATI	206 (75%)	214 (78%)	0.22	169 (77%)	174 (80%)	0.46	37 (67%)	40 (73%)	0.25
SMI									
Normal SMI	156 (57%)	137 (50%)		134 (62%)	119 (55%)		22 (40%)	18 (33%)	
Low SMI	117 (43%)	135 (50%)	**<0.01**	84 (38%)	99 (45%)	**0.01**	33 (60%)	36 (67%)	0.25
SMD									
Normal SMD	98 (36%)	33 (12%)		77 (36%)	24 (11%)		21 (38%)	9 (16%)	
Low SMD	175 (64%)	240 (88%)	**<0.01**	141 (64%)	194 (89%)	**<0.001**	34 (62%)	46 (84%)	**<0.001**

*Note*: *P* values generated through McNemar tests comparing the proportion of each covariate within each subgroup. Bold values denote statistically significant values (*P* < 0.05). Abbreviations: SATI, subcutaneous adipose tissue index; SMD, skeletal muscle density; SMI, skeletal muscle index; VATI, visceral adipose tissue index.

*Table* *S1* displays the sex‐specific differences between pre‐operative and follow‐up median (IQR) CT‐BC parameters in the entire longitudinal cohort (*n* = 273) and in patients subgrouped by statin therapy. Low absolute numbers (*n* = 1) of eligible female patients in the ‘no statin therapy’ subgroup precluded meaningful analysis of this subgroup. In the entire longitudinal cohort, from pre‐operative to follow‐up CTs, there was an increase in median SATI (*P* = 0.02) and VATI (*P* < 0.01) in male patients only; there was a decrease in median SMI (*P* < 0.001 in males, *P* < 0.01 in females) and SMD (*P* < 0.001 in males, *P* < 0.01 in females). In male patients taking statins (*P* = 0.04) and not taking statins (*P* = 0.02), there was an increase in VATI. There was a decrease in median SMI in patients both taking (*P* < 0.001 in males, *P* < 0.01 in females) and not taking (*P* < 0.001) statins. There was a decrease in median SMD in patients both taking (*P* < 0.001 in males, *P* < 0.01 in females) and not taking (*P* < 0.001) statins.

The comparison between patients with follow‐up CTs based on ≤10% loss of SMI (*n* = 208) and >10% loss of SMI (*n* = 65) and ≤10% loss of SMD (*n* = 98) and >10% loss of SMD (*n* = 175) is shown in *Table* *S2*. Low pre‐operative SMD was associated with a reduced rate of >10% loss of SMD (68% vs. 56%, *P* = 0.04), whilst other comparisons were not significant. *Table* *4* displays the association between baseline covariates and >10% loss of SMI or SMD as assessed by a univariate binary logistic regression model. Low pre‐operative SMD was associated with reduced odds of >10% SMD loss (odds ratio [OR] 0.59, 95% CI 0.35–0.99, *P* = 0.04), whilst other associations were non‐significant.

**Table 4:** The association between demographic and clinicopathological characteristics, CT‐BC, systemic inflammation and magnitude of SMI or SMD loss in patients undergoing elective EVAR or F/B‐EVAR for AAA subgrouped by statin therapy (*n* = 273)

	SMI loss > 10% (*n* = 65)	SMD loss > 10% (*n* = 175)
Age > 75 years	0.92	1.36
	0.61–1.38	0.95–1.95
	*P* = 0.69	*P* = 0.10
Female sex	0.84	1.23
	0.27–2.64	0.45–3.35
	*P* = 0.77	*P* = 0.69
ASA > 2	1.07	0.88
	0.61–1.87	0.54–1.46
	*P* = 0.82	*P* = 0.62
BMI ≥ 25 kg/m^2^	0.59	0.99
	0.31–1.14	0.53–1.86
	*P* = 0.12	*P* = 0.99
SIMD (more deprived)	1.02	1.18
	0.58–1.78	0.71–1.93
	*P* = 0.96	*P* = 0.53
AAA diameter ≥ 65 mm	0.83	1.05
	0.44–1.57	0.60–1.83
	*P* = 0.56	*P* = 0.87
Statin use	0.71	0.76
	0.36–1.37	0.40–1.43
	*P* = 0.31	*P* = 0.39
High baseline creatinine	0.98	1.14
	0.50–1.92	0.62–2.10
	*P* = 0.94	*P* = 0.67
Low baseline Hb	1.46	0.66
	0.71–3.00	0.34–1.28
	*P* = 0.31	*P* = 0.22
SIG ≥ 2	0.99	0.93
	0.65–1.51	0.65–1.34
	*P* = 0.97	*P* = 0.71
High pre‐operative SATI	0.89	2.00
	0.38–2.11	0.94–4.25
	*P* = 0.79	*P* = 0.07
High pre‐operative VATI	0.81	1.00
	0.43–1.51	0.56–1.77
	*P* = 0.50	*P* = 0.99
Low pre‐operative SMI	0.86	1.22
	0.49–1.51	0.74–2.01
	*P* = 0.59	*P* = 0.45
Low pre‐operative SMD	1.16	0.59
	0.65–2.06	0.35–0.99
	*P* = 0.62	*P* = **0.04**

*Note*: *P* values generated through univariate binary logistic regression analyses testing the association between each covariate and SMI or SMD loss; data presented as odds ratio, 95% confidence interval and *P* value. Bold values denote statistically significant values (*P* < 0.05). Abbreviations: AAA, abdominal aortic aneurysm; ASA, American Society of Anesthesiologists grade; BMI, body mass index; EVAR, endovascular aneurysm repair; F/B‐EVAR, fenestrated/branched EVAR; SATI, subcutaneous adipose tissue index; SIG, systemic inflammatory grade; SIMD, Scottish Index of Multiple Deprivation; SMD, skeletal muscle density; SMI, skeletal muscle index; VATI, visceral adipose tissue index.

**Figure 3:**
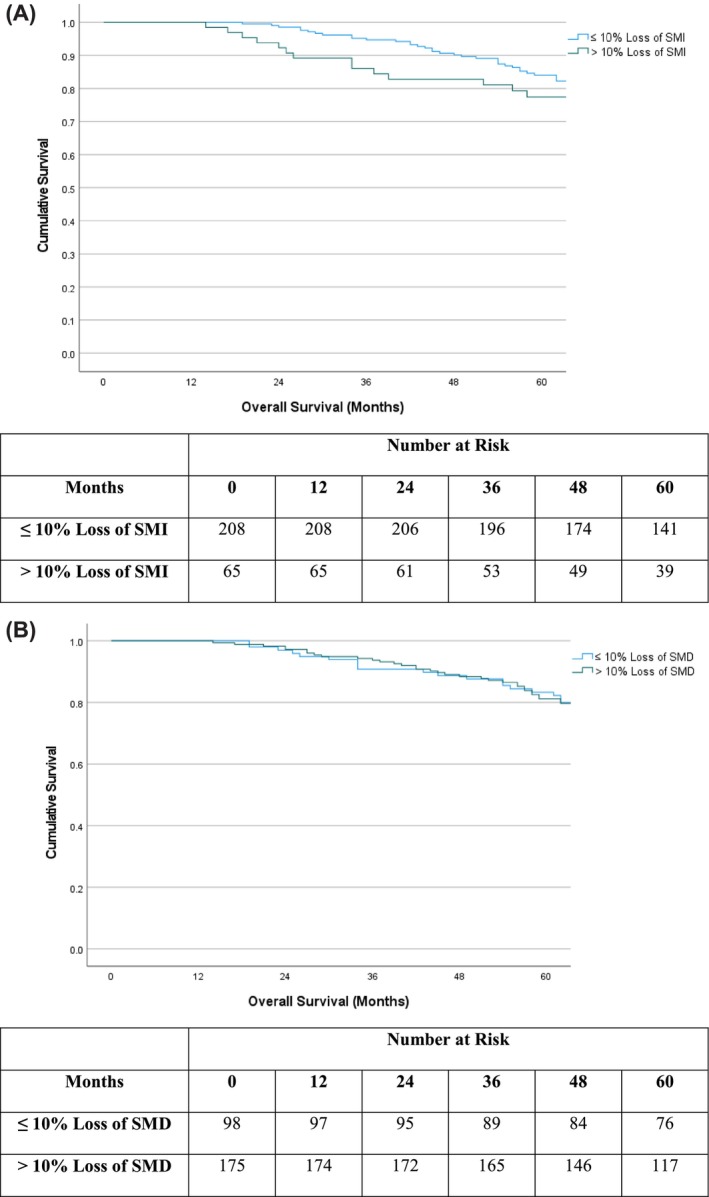
Kaplan–Meier survival plots and life table for loss (≤10% or >10%) of (A) skeletal muscle index (SMI; *P* = 0.58) and (B) skeletal muscle density (SMD; *P* = 0.56) between pre‐operative and follow‐up CT subgroups in patients undergoing elective endovascular repair of abdominal aortic aneurysm (*n* = 273).

*Figure* *3* displays Kaplan–Meier survival plots in patients subgrouped by ≤10% loss of SMI and >10% loss of SMI (*Figure* *3* *A*) and by ≤10% loss of SMD and >10% loss of SMD (*Figure* *3* *B*). In patients with ≤10% loss of SMI, mean (95% CI) survival was 91.6 (87.2–95.9) months versus 89.3 (80.4–98.2) months in patients with >10% loss of SMI (*P* = 0.58). In patients with ≤10% loss of SMD, mean (95% CI) survival was 89.5 (83.1–96.0) months versus 94.4 (89.3–99.5) months in patients with >10% loss of SMD (*P* = 0.56).

Scatter plots of change in CT‐BC parameters against follow‐up intervals (months) are shown in *Figures* *S1* and *S2*. For each CT‐BC parameter, the data presented do not show a linear relationship with time. A sensitivity analysis including only patients with a follow‐up interval from pre‐operative to follow‐up CTs of 11–18 months is shown in *Table* *S3*. In the sensitivity analysis, from pre‐operative to follow‐up CTs, there was a decrease in median SMI (*P* < 0.001) and SMD (*P* < 0.001) and an increase in the comparative prevalences of low SMI (41% vs. 47%, *P* = 0.04) and low SMD (67% vs. 87%, *P* < 0.001); however, there was no significant difference in the change of SATI or VATI.

## Discussion

The present study describes several important observations that contribute to the understanding of the complex interactions between body composition, statins and systemic inflammation. Skeletal muscle mass and density appear to continue to decline following repair of AAA, with significantly lower values observed at a relatively short interval from surgery. An association between statin therapy and greater pre‐operative skeletal muscle mass was observed; however, despite this association, statin therapy does not appear to be associated with the rate of loss of skeletal muscle following AAA repair. The typical pattern of change in body composition was a gain in subcutaneous and visceral fat with an associated loss in skeletal muscle mass and quality. This suggests the development of so‐called ‘sarcopenic obesity’, a clinical entity that may confer a discrete risk compared to high adiposity or low muscle mass in isolation.^24^, ^25^

The longitudinal data presented in the present study highlight the progressive nature of skeletal muscle loss, with the marked increase in prevalence of low SMD a particular novel finding of interest. These findings support those observed by Lindström et al.,^21^ though with the use of a more established CT‐BC methodology and assessment of the SIR. Despite the progressive changes in skeletal muscle observed, the present study did not observe a significant difference in mortality when patients were subgrouped based on the magnitude of muscle loss; however, this may reflect the relatively small sample size and hence the power of the study. A difference between the observations of the present study and those of Lindström et al. is that the former did not observe an association between statin therapy and an increased rate of muscle mass loss. Whilst this may reflect the modest sample size, this in particular highlights the complexity of the relationship between statin therapy and muscle tissue. Systemic inflammation appears to have a detrimental effect on skeletal muscle mass and density; indeed, Hacker et al. propose that low skeletal muscle mass should be considered a symptom of systemic inflammation rather than a discrete prognostic entity,^26^ and these observations have been reproduced in a cohort of patients with cardiovascular disease.^13^ Taken in conjunction with the evidence of statins as anti‐inflammatory agents, it is somewhat surprising that those patients taking statins in the present study did not display an increased rate of muscle loss. This perhaps highlights the dominant effect of host factors, for example, higher age, in determining skeletal muscle loss. Dolan et al. report that, in a cohort of 783 patients undergoing surgery for colorectal cancer, markers of systemic inflammation (mGPS) were associated with loss of skeletal muscle.^5^ The presence of colorectal cancer may, however, impact the validity of their results when discussing a non‐cancer cohort; we recently reported comparative data highlighting the increased magnitude of systemic inflammation in otherwise similar cohorts of patients with AAA and with colorectal cancer.^7^ Further analyses of longitudinal CTs in both of these cohorts are required.

The relationship between statin therapy and skeletal muscle has been described in prior studies. Valdiviesso et al. report a series of 136 patients with cardiac failure, where statin therapy was associated with a lower prevalence of clinically assessed sarcopenia.^20^ The present study reports similar observations in a different cohort of patients with cardiovascular disease, with the use of standardized methodology in CT‐BC analysis and the consideration of the SIR, which is an accepted major aetiological factor in the development of low skeletal muscle mass and sarcopenia.^27^ Myopathy as a result of statin therapy is well described, though there remains uncertainty as to the underlying mechanism.^18^, ^19^ This phenomenon makes the finding of higher skeletal muscle mass in the statin cohort somewhat unexpected. Another paradoxical observation is that this preservation of skeletal muscle occurred in a cohort with increased comorbidity and obesity, both of which have been associated with low skeletal muscle mass.^27^, ^28^ Interestingly, the association between low pre‐operative SMI and survival was more marked in the cohort of patients who did not receive a statin, in whom SMI itself was typically lower. It may be that as SMI declines, the effect on survival is not manifest until a ‘tipping point’ of muscle mass is reached, and that initially compensation for the loss of muscle mass occurs. Age, an important determinant of muscle mass, was consistently associated with inferior survival, independent of body composition, statin use or systemic inflammation.

Of particular interest, there was no reduction in the magnitude of the SIR in patients taking statins. The precise mechanism by which statins exert an anti‐inflammatory effect is incompletely understood, with multiple potential pathways suggested.^29^ It may be that SIG, used by the present study, does not capture the pathway involved, such as complement‐mediated inflammation, or that the present study was underpowered to observe an effect due to the relatively low prevalence of SIG ≥ 2 in patients with AAA (as compared to patients with cancer^9^). Regardless, the clinical benefit of statins appears to be greatest in patients with activation of the SIR, as observed in the JUPITER trial; Ridker et al. observed that, in patients with elevated high‐sensitivity CRP and low cholesterol, rosuvastatin therapy was associated with a reduced rate of cardiovascular events.^30^ Statins are of particular interest as a potential therapeutic agent, given their generally favourable side‐effect profile, cost and widespread availability. Whilst their use may mitigate the effect of systemic inflammation on prognosis, additional immunomodulatory adjuncts may also play a role in improving long‐term outcomes. A recent meta‐analysis reports that, in patients with atherosclerotic disease who are already receiving statin therapy, inflammation is the predominant factor influencing prognosis.^31^ Immunomodulation in atherosclerotic disease has been explored in large trials, with ongoing investigation to identify the agent with optimal efficacy and side‐effect profile.^32^, ^33^

The lack of data on drug dose and duration of therapy are potential sources of bias in the present study. It is likely that a proportion of patients in the ‘statin therapy’ subgroup were taking a dose below the maximal target; therefore, the population may be somewhat heterogeneous. Despite this, the direct association between statin therapy and maintained skeletal muscle mass was observed, indicating that, should the study be repeated with all patients on maximal‐dose statin, the ‘statin effect’ would manifest to a greater extent. This is an important area of further investigation.

## Limitations

The present study is limited by a retrospective study design, missing data for some participants, a wide range of follow‐up intervals and the use of data‐derived thresholds for CT‐BC analysis, which are a potential source of bias and require external validation. Despite missing longitudinal data for some patients, a strength of the use of protocolized follow‐up CTs is the ability to exclude potentially confounding diagnoses; CTs performed outside of protocol will be subject to potential bias due to the unknown effect on the parameters of interest. An additional weakness of the present study is the wide range of intervals from procedure to follow‐up CTs (range 12–71 months); however, normalization and sensitivity analyses were performed to attempt to mitigate this source of bias. CT surveillance protocols were limited by a loss of follow‐up, and in some cases, access to CT scanning was disrupted due to the Covid‐19 pandemic. Low absolute numbers in some subgroups limit the validity of some conclusions drawn. The conclusions would be strengthened by data on the duration and dose of statin therapy; however, these data were not available. Additionally, data on the type of statin (e.g., hydrophilic vs. hydrophobic) prescribed were not available, which is another potential source of bias. Specific causes of death may strengthen our assumptions regarding increased cardiovascular morbidity and mortality. We cannot exclude the role of selection bias in our cohort. It may be that the patients who did not take statin in the present study had previously been intolerant of statin therapy, indicative of a specific phenotype that is predisposed to statin myopathy. Therefore, the results may not be generalizable to a wider, unselected population. Finally, in the present analysis, 93% of the patients studied were male, and therefore, a separate analysis of males and females was not carried out given the likely minimal impact of females on the results obtained. It would be of interest to examine the present relationships in a cohort of female patients.

## Conclusions

In patients with AAA, skeletal muscle mass and density appear to progressively decline despite treatment of AAA, though specific determinants of this are uncertain, and statin use does not appear to predispose to either muscle loss or preservation. Statin therapy appears to be associated with a lower rate of pre‐operative low skeletal muscle mass, despite greater comorbidity and BMI. Further investigation of the mechanistic pathways between progressive changes in muscle mass and quality, statin therapy and systemic inflammation is warranted.

## Conflict of interest statement

N. A. Bradley, A. Walter, C. Sankey, A. Wilson, T. Siddiqui, C. S. D. Roxburgh, D. C. McMillan and G. J. K. Guthrie have no conflict of interest to declare.

## Supporting information


**Figure S1.** Scatter plots of total percentage change of A) Subcutaneous Adipose Tissue Index (ΔSATI) and B) Visceral Adipose Tissue Index (ΔVATI) against number of months between pre‐operative and follow‐up CTs in patients undergoing elective endovascular repair of abdominal aortic aneurysm (*n* = 273).

**Figure S2.** Scatter plots of total percentage change of A) Skeletal Muscle Index (ΔSMI) and B) Skeletal Muscle Density (ΔSMD) against number of months between pre‐operative and follow‐up CTs in patients undergoing elective endovascular repair of abdominal aortic aneurysm (*n* = 273).

**Table S1.** The comparison of pre‐operative and longitudinal CT‐derived body composition parameters (continuous) in patients with per protocol follow‐up CTs undergoing elective EVAR or F/B‐EVAR for AAA, sub‐grouped by statin use (*n* = 273).
**Table S2.** The comparison of demographic and clinicopathological characteristics, CT‐BC, and systemic inflammation between patients with >10% and ≤ 10% loss of SMI or SMD on follow‐up CT in a cohort of patients undergoing elective EVAR or F/B‐EVAR for AAA (*n* = 273).
**Table S3.** The comparison of pre‐operative and longitudinal CT‐derived body composition parameters in patients with per protocol follow‐up CTs undergoing elective EVAR or F/B‐EVAR for AAA, sensitivity analysis with follow‐up interval ≥ 11 and ≤ 18 months (*n* = 180).
