# Supplementary material for: Longitudinal association between statins and changes in CT‐derived body composition in patients with abdominal aortic aneurysm
Source: J Cachexia Sarcopenia Muscle. 2025 Apr 9;16(2):10.1002/jcsm.13565. doi: 10.1002/jcsm.13565 (PMC11981686; doi:10.1002/jcsm.13565)
Supplement: Supplementary file 1 — Figure S1. Scatter plots of total percentage change of A) Subcutaneous Adipose Tissue Index (ΔSATI) and B) Visceral Adipose Tissue Index (ΔVATI) against number of months between pre‐operative and follow‐up CTs in patients undergoing elective endovascular repair of abdominal aortic aneurysm (n = 273). [file JCSM-16--s003.docx]

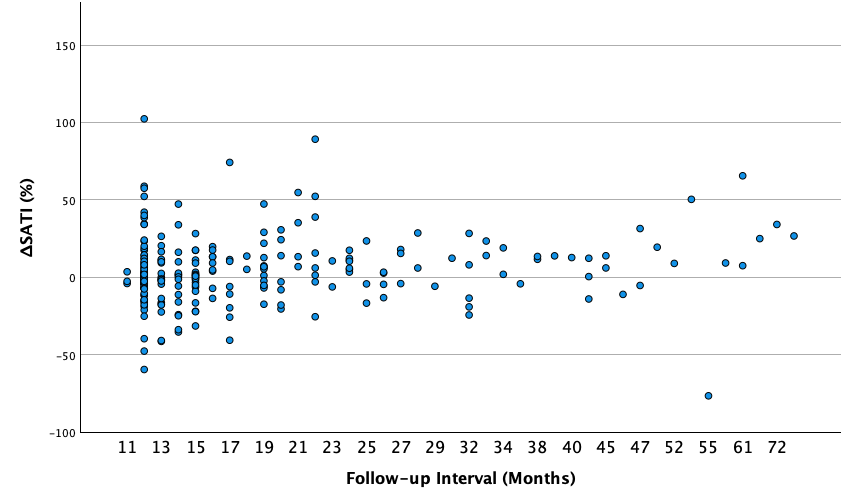


**A)**

**B)**


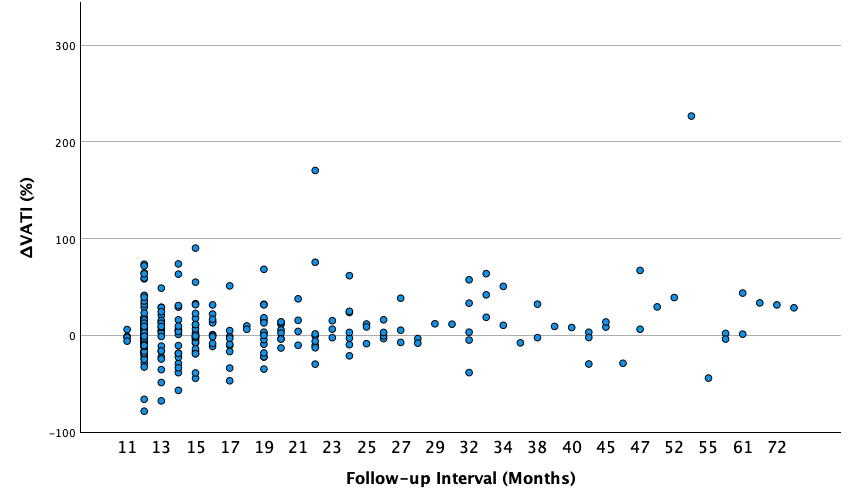


**Supplemental Fig. 1** Scatter plots of total percentage change of A) Subcutaneous Adipose Tissue Index (ΔSATI) and B) Visceral Adipose Tissue Index (ΔVATI) against number of months between pre-operative and follow-up CTs in patients undergoing elective endovascular repair of abdominal aortic aneurysm (n = 273)
