# Supplementary material for: Longitudinal association between statins and changes in CT‐derived body composition in patients with abdominal aortic aneurysm
Source: J Cachexia Sarcopenia Muscle. 2025 Apr 9;16(2):10.1002/jcsm.13565. doi: 10.1002/jcsm.13565 (PMC11981686; doi:10.1002/jcsm.13565)

**Supplemental Fig. 2** Scatter plots of total percentage change of A) Skeletal Muscle Index (ΔSMI) and B) Skeletal Muscle Density (ΔSMD) against number of months between pre-operative and follow-up CTs in patients undergoing elective endovascular repair of abdominal aortic aneurysm (n = 273)

**B)**

**A)**


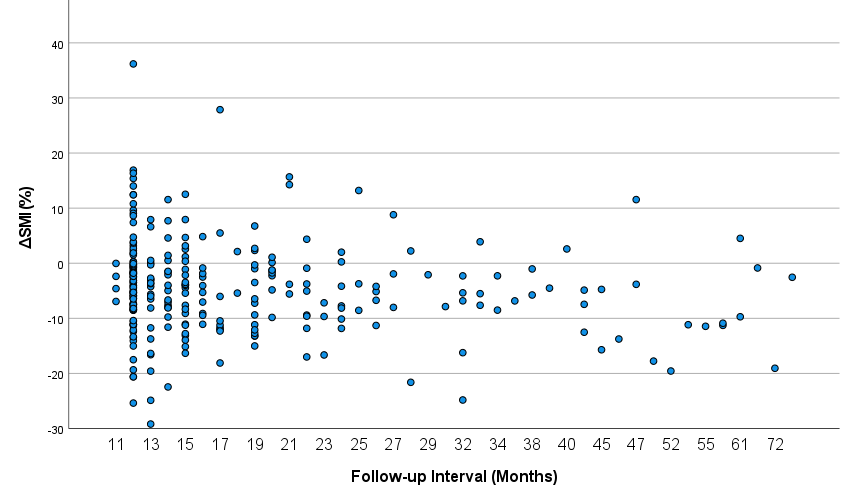

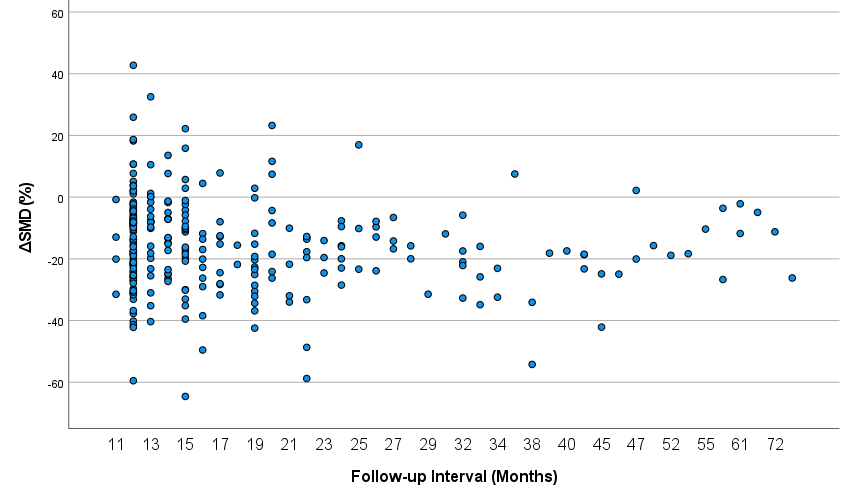

Supplement: Supplementary file 2 — Figure S2. Scatter plots of total percentage change of A) Skeletal Muscle Index (ΔSMI) and B) Skeletal Muscle Density (ΔSMD) against number of months between pre‐operative and follow‐up CTs in patients undergoing elective endovascular repair of abdominal aortic aneurysm (n = 273). [file JCSM-16--s002.docx]
