# Supplementary material for: Longitudinal association between statins and changes in CT‐derived body composition in patients with abdominal aortic aneurysm
Source: J Cachexia Sarcopenia Muscle. 2025 Apr 9;16(2):10.1002/jcsm.13565. doi: 10.1002/jcsm.13565 (PMC11981686; doi:10.1002/jcsm.13565)
Supplement: Supplementary file 3 — Table S1. The comparison of pre‐operative and longitudinal CT‐derived body composition parameters (continuous) in patients with per protocol follow‐up CTs undergoing elective EVAR or F/B‐EVAR for AAA, sub‐grouped by statin use (n = 273). Table S2. The comparison of demographic and clinicopathological characteristics, CT‐BC, and systemic inflammation between patients with >10% and ≤ 10% loss of SMI or SMD on follow‐up CT in a cohort of patients undergoing elective EVAR or F/B‐EVAR for AAA (n = 273). Table S3. The comparison of pre‐operative and longitudinal CT‐derived body composition parameters in patients with per protocol follow‐up CTs undergoing elective EVAR or F/B‐EVAR for AAA, sensitivity analysis with follow‐up interval ≥ 11 and ≤ 18 months (n = 180). [file JCSM-16--s001.docx]

| **Supplemental Table 1:** The comparison of pre-operative and longitudinal CT-derived body composition parameters (continuous) in patients with per protocol follow-up CTs undergoing elective EVAR or F/B-EVAR for AAA, sub-grouped by statin use (n = 273). | | | | | | | | | |  |
| --- | --- | --- | --- | --- | --- | --- | --- | --- | --- | --- |
|  | **Entire Longitudinal Cohort (n = 273)** | | | **Statin Therapy (n = 218)** | | | **No Statin Therapy (n = 55)** | | |  |
|  | **Pre-operative CT** | **Follow-up CT** | ***p*** | **Pre-operative CT** | **Follow-up CT** | ***p*** | **Pre-operative CT** | **Follow-up CT** | ***p*** |  |
| **SATI** |  |  |  |  |  |  |  |  |  |  |
| Median (IQR) | 63.6 (37.5) cm^2^/m^2^ | 66.9 (38.6) cm^2^/m^2^ | **0.03** | 64.7 (39.1) cm^2^/m^2^ | 66.9 (36.8) cm^2^/m^2^ | 0.06 | 59.8 (36.1) cm^2^/m^2^ | 65.7 (41.3) cm^2^/m^2^ | 0.14 |  |
| Median (IQR), Males | 62.3 (33.8) | 65.9 (36.4) cm^2^/m^2^ | **0.03** | 62.6 (33.0) cm^2^/m^2^ | 65.9 (35.7) cm^2^/m^2^ | 0.08 | 59.8 (36.0) cm^2^/m^2^ | 67.6 (39.8) cm^2^/m^2^ | 0.16 |  |
| Median (IQR), Females | 96.5 (48.6) cm^2^/m^2^ | 101.7 (53.7) cm^2^/m^2^ | 0.35 | 98.5 (35.6) cm^2^/m^2^ | 101.7 (33.2) cm^2^/m^2^ | 0.39 | - | - | - |  |
| **VATI** |  |  |  |  |  |  |  |  |  |  |
| Median (IQR) | 83.0 (55.5) cm^2^/m^2^ | 87.6 (56.1) cm^2^/m^2^ | **<0.01** | 82.8 (52.2) cm^2^/m^2^ | 85.3 (53.7) cm^2^/m^2^ | **0.04** | 83.5 (64.8) cm^2^/m^2^ | 88.4 (60.5) cm^2^/m^2^ | **0.02** |  |
| Median (IQR), Males | 86.0 (56.9) cm^2^/m^2^ | 88.9 (57.1) cm^2^/m^2^ | **<0.01** | 86.0 (51.1) cm^2^/m^2^ | 88.9 (57.0) cm^2^/m^2^ | **0.04** | 84.9 (63.1) cm^2^/m^2^ | 89.2 (59.9) cm^2^/m^2^ | **0.02** |  |
| Median (IQR), Females | 61.4 (48.0) cm^2^/m^2^ | 66.4 (48.4) cm^2^/m^2^ | 0.78 | 66.4 (44.3) cm^2^/m^2^ | 66.5 (48.2) cm^2^/m^2^ | 0.65 | - | - | - |  |
| **SMI** |  |  |  |  |  |  |  |  |  |  |
| Median (IQR) | 49.9 (12.7) cm^2^/m^2^ | 47.7 (12.7) cm^2^/m^2^ | **<0.001** | 51.2 (12.6) cm^2^/m^2^ | 48.8 (13.1) cm^2^/m^2^ | **<0.001** | 45.8 (11.4) cm^2^/m^2^ | 43.5 (12.1) cm^2^/m^2^ | **<0.001** |  |
| Median (IQR), Males | 60.0 (12.8) cm^2^/m^2^ | 48.4 (12.7) cm^2^/m^2^ | **<0.001** | 52.1 (11.6) cm^2^/m^2^ | 50.2 (11.2) cm^2^/m^2^ | **<0.001** | 45.8 (11.6) cm^2^/m^2^ | 43.8 (12.0) cm^2^/m^2^ | **<0.001** |  |
| Median (IQR), Females | 39.9 (7.2) cm^2^/m^2^ | 38.7 (9.0) cm^2^/m^2^ | **<0.01** | 40.0 (6.5) cm^2^/m^2^ | 39.1 (8.9) cm^2^/m^2^ | **<0.01** | - | - | - |  |
| **SMD** |  |  |  |  |  |  |  |  |  |  |
| Median (IQR) | 37.4 (10.1) HU | 31.2 (10.2) HU | **<0.001** | 37.1 (10.1) HU | 31.0 (10.2) HU | **<0.001** | 39.7 (10.6) HU | 32.1 (10.7) HU | **<0.001** |  |
| Median (IQR), Males | 38.0 (10.3) HU | 31.8 (9.9) HU | **<0.001** | 37.4 (10.2) HU | 31.3 (9.6) HU | **<0.001** | 39.9 (10.6) HU | 32.0 (11.1) HU | **<0.001** |  |
| Median (IQR), Females | 31.9 (9.3) HU | 24.0 (9.0) HU | **<0.01** | 31.7 (7.0) HU | 23.6 (8.4) HU | **<0.001** | - | - | - |  |
| *p* values generated through Wilcoxon matched-pairs tests. Low absolute numbers (n = 1) of eligible female patients in the “No Statin Therapy” sub-group precluded meaningful analysis.  IQR: interquartile range. SATI: subcutaneous adipose tissue index. VATI: visceral adipose tissue index. SMI: skeletal muscle index. SMD: skeletal muscle density | | | | | | | | | | |

| **Supplemental Table 2:** The comparison of demographic and clinicopathological characteristics, CT-BC, and systemic inflammation between patients with >10% and ≤ 10% loss of SMI or SMD on follow-up CT in a cohort of patients undergoing elective EVAR or F/B-EVAR for AAA (n = 273). | | | | | | | |
| --- | --- | --- | --- | --- | --- | --- | --- |
|  | **>10% Loss of SMI (n = 65)** | **≤10% Loss of SMI (n = 208)** | ***p*** | **>10% Loss of SMD (n = 175)** | **≤10% Loss of SMD (n = 98)** | ***p*** |  |
| **Age**  < 65  65 - 75  > 75 | 3 (5%)  37 (57%)  25 (38%) | 13 (6%)  114 (55%)  81 (39%) | 0.88 | 18 (10%)  71 (41%)  86 (49%) | 15 (15%)  44 (45%)  39 (40%) | 0.10 |  |
| **Sex**  Male  Female | 61 (94%)  4 (6%) | 193 (93%)  15 (7%) | 0.77 | 162 (93%)  13 (7%) | 92 (94%)  6 (6%) | 0.69 |  |
| **ASA**  ≤ 2  > 2 | 36 (55%)  29 (45%) | 118 (57%)  89 (43%) | 0.82 | 101 (58%)  74 (42%) | 53 (55)  44 (45%) | 0.63 |  |
| **BMI**  < 25 kg/m^2^  ≥ 25 kg/m^2^ | 17 (26%)  48 (74%) | 36 (17%)  172 (83%) | 0.12 | 34 (19%)  141 (81%) | 19 (19%0  79 (81%) | 0.99 |  |
| **SIMD**  More Deprived  Less Deprived | 28 (43%)  37 (57%) | 90 (44%)  117 (56%) | 0.96 | 73 (42%)  101 (58%) | 45 (46%)  53 (54%) | 0.53 |  |
| **Baseline AAA Diameter**  <65mm  ≥ 65mm | 49 (75%)  16 (25%) | 147 (72%)  58 (28%) | 0.56 | 125 (72%)  48 (28%) | 71 (73%)  26 (27%) | 0.87 |  |
| **Statin Use**  Yes  No | 49 (75%)  16 (25%) | 169 (81%)  39 (19%) | 0.30 | 137 (78%)  28 (22%) | 81 (83%)  17 (17%) | 0.39 |  |
| **Baseline Creatinine**  Normal  High | 51 (79%)  14 (21%) | 157 (77%)  48 (23%) | 0.75 | 133 (77%)  39 (23%) | 78 (80%)  20 (20%) | 0.76 |  |
| **Baseline Haemoglobin**  Normal  Low | 52 (80%)  13 (20%) | 175 (85%)  30 (15%) | 0.30 | 149 (86%)  24 (14%) | 78 (80%)  19 (20%) | 0.22 |  |
| **SIG**  0  1  ≥ 2 | 31 (57%)  15 (28%)  8 (15%) | 103 (56%)  57 (31%)  25 (13%) | 0.97 | 85 (57%)  44 (30%)  20 (13%) | 49 (54%)  28 (31%)  13 (15%) | 0.71 |  |
| **Pre-operative SATI**  Normal  High | 8 (13%)  53 (87%) | 23 (12%)  171 (88%) | 0.79 | 15 (9%)  146 (91%) | 16 (17%)  78 (83%) | 0.07 |  |
| **Pre-operative VATI**  Normal  High | 18 (28%)  47 (72%) | 49 (24%)  159 (76%) | 0.50 | 43 (25%)  132 (75%) | 24 (25%)  74 (75%) | 0.99 |  |
| **Pre-operative SMI**  Normal  Low | 39 (60%)  26 (40%) | 117 (56%)  91 (44%) | 0.60 | 97 (55%)  78 (45%) | 59 (60%)  39 (40%) | 0.45 |  |
| **Pre-operative SMD**  Normal  Low | 22 (34%)  43 (66%) | 76 (37%)  132 (63%) | 0.69 | 77 (44%)  98 (56%) | 31 (32%)  67 (68%) | **0.04** |  |
| *p* values generated through linear-by-linear Chi Squared analyses comparing proportion of each covariate within each subgroup.  ASA: American society of anaesthesiologists grade. BMI: body mass index. SIMD: Scottish index of multiple deprivation. EVAR: endovascular aneurysm repair. F/B-EVAR: fenestrated/branched EVAR. AAA: abdominal aortic aneurysm. SIG: systemic inflammatory grade. SATI: subcutaneous adipose tissue index. VATI: visceral adipose tissue index. SMI: skeletal muscle index. SMD: skeletal muscle density | | | | | | | |

|  | | | |
| --- | --- | --- | --- |
| **Supplemental Table 3:** The comparison of pre-operative and longitudinal CT-derived body composition parameters in patients with per protocol follow-up CTs undergoing elective EVAR or F/B-EVAR for AAA, sensitivity analysis with follow-up interval ≥ 11 and ≤ 18 months (n = 180). | | | |
|  | **Pre-operative CT (n = 180)** | **Follow-up CT (n = 180)** | ***p*** |
| **SATI**  Median (IQR)  Normal SATI  High SATI | 65.5 (40.2) cm^2^/m^2^  20 (12%)  146 (88%) | 67.1 (38.1) cm^2^/m^2^  25 (15%)  147 (85%) | 0.89  0.11 |
| **VATI**  Median (IQR)  Normal VATI  High VATI | 88.8 (60.1) cm^2^/m^2^  41 (23%)  139 (77%) | 89.9 (57.4) cm^2^/m^2^  38 (21%)  142 (79%) | 0.28  0.66 |
| **SMI**  Median (IQR)  Normal SMI  Low SMI | 51.0 (12.4) cm^2^/m^2^  107 (59%)  73 (41%) | 48.6 (12.9) cm^2^/m^2^  95 (53%)  84 (47%) | **<0.001**  **0.04** |
| **SMD**  Median (IQR)  Normal SMD  Low SMD | 37.1 (9.7) HU  60 (33%)  120 (67%) | 32.1 (11.3) HU  23 (13%)  157 (87%) | **<0.001**  **<0.001** |
| *p* values generated through Wilcoxon matched-pairs tests for continuous data and through McNemar tests comparing proportion of each covariate within each subgroup for categorical data.  IQR: interquartile range. SATI: subcutaneous adipose tissue index. VATI: visceral adipose tissue index. SMI: skeletal muscle index. SMD: skeletal muscle density | | | |
